# The Combined Use of Platelet-Rich Plasma Clot Releasate and Allogeneic Human Umbilical Cord Mesenchymal Stem Cells Rescue Glucocorticoid-Induced Osteonecrosis of the Femoral Head

**DOI:** 10.1155/2022/7432665

**Published:** 2022-05-02

**Authors:** Yanxue Wang, Shuo Luan, Ze Yuan, Shaoling Wang, Shengnuo Fan, Chao Ma, Shaoling Wu

**Affiliations:** Department of Rehabilitation Medicine, Sun Yat-sen Memorial Hospital, Sun Yat-sen University, Guangzhou, Guangdong, China

## Abstract

Glucocorticoid-induced osteonecrosis of the femoral head (ONFH) is a refractory disease. The treatment options for ONFH, especially nonsurgical ones, merit further investigation. To evaluate the combinatorial therapeutic effects of platelet-rich plasma clot releasate (PRCR) and umbilical cord mesenchymal stem cells (UC-MSCs) on glucocorticoid-induced ONFH, a dexamethasone (DEX)-treated cell model and a high-dose methylprednisolone (MPS)-treated rat model were established. Cell counting kit-8 (CCK-8) assay was performed *in vitro* to determine the optimum dosage of PRCR for UC-MSC viability. The effects of PRCR, UC-MSCs, and PRCR + UC-MSCs on cell viability, apoptosis, migration, and differentiation capacities of DEX-treated bone marrow mesenchymal stem cells (BMSCs) and human umbilical vein endothelial cell (HUVECs) were explored via Transwell assays. Western blotting was conducted to evaluate the expression levels of RUNX2, VEGF, caspase-3, and Bcl-2 in the coculture systems. Ultrasound-guided intra-articular PRCR, UC-MSCs, and PRCR + UC-MSC injections were performed on the ONFH model rats. Microcomputed tomography, histological and immunohistochemical analyses, tartrate-resistant acid phosphatase (TRAP) staining, and terminal deoxynucleotidyl transferase dUTP nick end labeling (TUNEL) staining were used to assess the therapeutic effects of PRCR and UC-MSCs on bone loss and necrosis induced by high-dose MPS. Results of this study revealed that the *in vitro* application of PRCR, UC-MSCs, and PRCR + UC-MSCs reversed the impaired proliferation and migration capacities and resisted apoptosis of BMSCs and HUVECs induced by DEX. Moreover, the PRCR and UC-MSC application significantly improved the alkaline phosphatase (ALP) and alizarin red (ALR) staining of BMSCs and tube formation capacity of HUVECs and promoted the protein expression of RUNX2 in BMSCs and VEGF in HUVECs. Similarly, in the ONFH rat model, the intra-articular injection of UC-MSCs and PRCR improved the subchondral bone mass parameters; promoted the expression of ALP, RUNX2, and VEGF; suppressed osteoclast overactivity; and resisted cell apoptosis. The combination of PRCR and UC-MSCs shows promising therapeutic effects in treating glucocorticoid-induced ONFH. The current study provides important information on intra-articular therapy, paving the way for the clinical management of ONFH in the future.

## 1. Introduction

In recent years, the incidence of osteonecrosis of the femoral head (ONFH) has gradually increased, affecting more and more younger individuals. ONFH is devastating and progressive and usually leads to the loss of physical and social function [1]. More than 60% of symptomatic ONFH cases progress rapidly; thus, these young and middle-aged patients might require total hip arthroplasties (THA) and even revision arthroplasty during their later life [2]. High-dose glucocorticosteroid-induced ONFH is one of the common causes of nontraumatic ONFH [3]. Regional trabecular bone fracture, subchondral bone necrosis, and empty osteocyte lacunae are typical pathological changes observed in the necrotic zones of the femoral head. If not treated promptly, ONFH will progress to a stage where the affected hip joint experiences extensive collapse and degeneration [4]. Glucocorticoid-induced local thrombi and fat emboli might be the main causes of vascular occlusion and endothelial injury. The decreased blood flow and ischemia further lead to subsequent osteogenesis impairment, including abnormal osteoprogenitor cell differentiation [5]. In healthy individuals, local osteoprogenitor cells which exist in the proximal femur usually play crucial roles in bone formation and the natural repair process. These stem cells are generally activated by physiological stimulation and subsequently permit proliferation, osteogenic differentiation, and extracellular matrix mineralization potentials. However, the natural repair potential tends to be impaired under certain severe conditions, such as high-dose corticosteroid application. Recent studies have confirmed the presence of functionally defective stem cells within the necrotic zone of diagnosed glucocorticosteroid-induced ONFH patients [6].

For patients with late-stage ONFH, the inevitable therapy choice is THA, and the revision arthroplasty is often required later on since the lifespan of the prosthesis is approximately 15 years. Current intervention options, either surgical or nonsurgical, are still not satisfactory [7]. The MSCs belong to a category of multipotent stem cells with low immunogenicity and multidirectional differentiation potential, making MSCs a promising candidate in regenerative biologics [8, 9]. It has been shown that MSCs secrete active growth factors and immunomodulatory cytokines. An increasing number of studies in the past decades have confirmed the positive therapeutic effects of MSCs on musculoskeletal diseases [10, 11].

In the present study, the allogeneic umbilical cord mesenchymal stem cells (UC-MSCs) were adopted for the following reasons. Firstly, bone marrow MSCs (BMSCs) have already been studied in basic and clinical ONFH research [12, 13]. However, there are some inevitable disadvantages of BMSCs. The procedure of BMSCs harvesting is usually painful, invasive, and complicated. UC-MSCs, in contrast, are derived from Wharton's jelly and usually procured in a noninvasive manner before the umbilical cord is discarded after a child's birth. This is comparatively easy, safe, and ethically feasible [14]. Secondly, UC-MSCs can be isolated with high efficiency and possess higher proliferative capacity than BMSCs and adipose-derived mesenchymal stem cells (ADSCs). Moreover, UC-MSCs exhibit lower immunogenicity than MSCs from other sources, so a complete human leukocyte antigen (HLA) match is not required [15]. Allogeneic human UC-MSCs have been widely used in the clinical management of many diseases, such as bone defects, spinal cord injury, and osteoarthritis [16–18]. Nevertheless, few studies have addressed the roles and mechanisms of allogeneic UC-MSCs in treating ONFH via *in vivo* models [19].

Platelet-rich plasma (PRP) is an autologous blood bioproduct composed of concentrated platelets and growth factors, such as transforming growth factor-*β* (TGF-*β*), insulin-like growth factor (IGF), platelet-derived growth factor (PDGF), and vascular endothelial growth factor (VEGF). By releasing a pool of growth factors and cytokines from a-granules after platelet activation, platelet-rich clot releasate (PRCR) helps to create a prohealing microenvironment in injured tissues [20]. Emerging evidence suggests that PRP formulation (including the PRCR or PRP lysate) yields superior regenerative and reconstructive outcomes in treating ONFH, either through intra-articular injection or transplantation combined with core decompression [21, 22]. Despite the strengths of PRP treatment, more effective options are needed for patients with advanced degeneration and injuries. Previous research has demonstrated that PRP preserves the immune-privileged properties and delays the appearance of senescent MSCs [23]. Furthermore, PRP was shown to enhance the proliferation and differentiation of MSCs, supporting the rationale that combined administration of PRP and MSCs might achieve synergistic effects [24]. For example, Wen et al. reported that PRP promoted the proliferation and differentiation of UC-MSCs within in *vitro* studies besides confirming that the combination of PRP with UC-MSCs could restore early stage bone defects in a rat model [25]. However, whether the combined use of PRP formulation and UC-MSCs can prevent the progression of ONFH and provide synergistic regenerative effects in glucocorticoid-induced ONFH remains to be explored.

In this present study, we established *in vitro and in vivo* models to mimic the pathophysiology process of glucocorticosteroid-induced ONFH. High dosage of dexamethasone caused deleterious effects on BMSCs and human umbilical vein endothelial cells (HUVECs), and the high dosage of MPS administrated to rodents served to directly mimic the femoral head necrosis pathological changes [26, 27]. Moreover, the *in vitro* efficacy of a combined treatment of PRCR plus UC-MSCs on the proliferation, migration, and differentiation of BMSCs and human umbilical vein endothelial cells (HUVECs) was examined. Additionally, an ONFH rat model was established to compare the therapeutic effects of PRCR and UC-MSCs combined, as well as the use of PRCR or UC-MSCs alone via intra-articular injection. It is hopeful that the biological therapies explored in this study might pave the road for the future clinical practice of ONFH.

## 2. Materials and Methods

### 2.1. *In Vitro* Experiments

#### 2.1.1. Platelet-Rich Plasma Clot Releasate (PRCR) Preparation and Growth Factor Quantification

Platelet-rich plasma was collected from apheresis platelets provided by the Blood Transfusion Department of Sun Yat-sen Memorial Hospital, and approval was obtained from the ethics committee of Sun Yat-sen Memorial Hospital (2021(429)). To ensure the homogeneity of collected PRP, the apheresis platelets were collected from the platelet-plasma mixture from a single healthy male donor aged 35. 10% calcium chloride (CaCl_2,_ Sigma-Aldrich) plus 1000 U/mL thrombin from bovine plasma (T8021, Solarbio, Beijing, China) were added to the apheresis platelets at a ratio of 1 : 10 for activation [28]. The mixture was incubated at 37°C for one hour and then at 4°C for 12 hours. After that, the mixture was centrifuged at 2800 × g and 4°C for 25 min, after which the supernatant soluble PRCR was isolated from the clotted sediment. The final PRCR was filtered through 0.22 *μ*m filters via ultrafiltration and was then aliquoted and stored at -80°C for further use. All procedures were performed under aseptic conditions. Enzyme-linked immunosorbent assay (ELISA) was conducted using a Human Vascular Endothelial Growth Factor A (VEGF-A) ELISA Kit (RayBiotech, Guangzhou) and a Human Transforming Growth Factor beta 1 (TGF-*β*1) ELISA Kit (RayBiotech, Guangzhou) to detect the concentrations of the growth factors VEGF-A and TGF-*β*1.

#### 2.1.2. Cell Culture

The single-donor human BMSCs and human UC-MSCs used in this study were purchased from Guangzhou SALIAI Stem Cell Co., Ltd. and were shown to express a variety of stem cell-specific markers, indicating good potential for proliferation and multidifferentiation. The single donor HUVEC cell line c-12206 was procured from PromoCell, Germany. The BMSCs, UC-MSCs, and HUVECs were seeded in cell culture flasks (Corning, New York, USA) before being cultured in the complete medium: Dulbecco's modified Eagle's medium-low glucose (DMEM-LG, Gibco, USA) containing 10% fetal bovine serum (Gibco, Australia) and 1% penicillin-streptomycin solution (Gibco, USA), at a seeding density of 5 × 10^4^ cells/cm^2^. The cells were cultured in a humidified incubator supplemented with 5% CO_2_ at 37°C, and the medium was changed every three days. After growing to 80% confluence, the BMSCs, UC-MSCs, and HUVECs were detached by 0.25% trypsin (Solarbio, Beijing). Then, the complete medium was added to stop the digestion, and centrifugation was performed at 250 × g for five min, after which the supernatant was discarded. The fresh medium was added for passage and cultured with a density of 2 × 10^5^ cells in 25 cm^2^ cell culture flasks (Corning). BMSCs, UC-MSCs, and HUVECs were used between passages 3 and 5 in the follow-up experiments. In addition, the BMSCs and HUVECs were treated with dexamethasone (DEX, Sigma-Aldrich, USA) to establish a cell model *in vitro*.

#### 2.1.3. Preliminary Study: Optimal Concentrations of PRCR on UC-MSC Proliferation

The optimal concentration for promoting stem cell proliferation varies in different studies with different methods of PRP preparation and platelet contents [24, 25]. To determine the optimal dosage of PRCR on UC-MSC proliferation, a cell counting kit-8 (CCK-8) cell viability assay was performed as the preliminary study. UC-MSCs were seeded in 96-well plates (Corning, USA) at 5 × 10^3^ cells/well, and the wells without cells served as the blank group. The cells were divided into six groups with three replicates per group and were treated with corresponding PRCR concentrations: (1) control group (cultured with complete medium, without PRCR treatment), (2) 0.5% PRCR group, (3) 1% PRCR group, (4) 2% PRCR group, (5) 4% PRCR group, and (6) 5% PRCR group. At 24 hours, 48 hours, and 72 hours, 10 *μ*L of CCK-8 reagent (ApexBio Technology, USA) was added into 100 *μ*L of the culture medium in each well and then coincubated for one hour at 37°C. The optical density (OD) was read at a wavelength of 450 nm using the microplate reader from Thermo Fisher Scientific (USA) to evaluate the proliferation rate of UC-MSCs.

#### 2.1.4. Coculture Assay

To evaluate the therapeutic effects of PRCR, UC-MSC, and PRCR + UC-MSC treatments on BMSCs and HUVECs, two coculture systems were established. The concentration of DEX was 10 *μ*M in the proliferation experiment, migration experiment, osteogenic differentiation experiment, and tube formation experiment, and 100 *μ*M was used in the apoptosis experiment, because DEX is more likely to induce cell apoptosis at a relatively high dosage [29]. Transwell plates were purchased from Corning (USA); the 8 *μ*m pore size Transwell system was used in cell migration experiment and the 0.4 *μ*m pore size in cell proliferation assay, apoptosis assay, BMSC osteogenic differentiation, and HUVEC tube formation assays.

*(1) Proliferation Assay*. UC-MSCs and BMSCs/HUVECs were cocultured in noncontact Transwell systems to assess the effects of PRCR and UC-MSCs on BMSC/HUVEC proliferation. Five different groups of interventions were analyzed: (1) control group, (2) DEX group (treated with DEX), (3) PRCR group (treated with DEX+2% PRCR), (4) UC-MSC group (treated with DEX + UC-MSCs), and (5) PRCR + UC-MSC group (treated with DEX+2% PRCR + UC-MSCs). The coculture inserts (0.4 *μ*m pores) were placed in 24-well plates. BMSCs/HUVECs were added to the lower culture wells (4 × 10^4^ cells per well), whereas UC-MSC cells (2 × 10^4^ cells per well) were placed in the inserts and cultured in the complete medium for both the UC-MSC and PRCR + UC-MSC groups. The CCK-8 assay was performed according to the manufacturer's protocol. On days 2 and 4, 50 *μ*L of CCK-8 solution was added to the BMSCs/HUVECs in each well and incubated at 37°C for one hour. The OD values of all five groups were read at 450 nm.

*(2) Migration Assay*. BMSC/HUVEC migration assays were performed using 24-well Transwell plates, with groupings as mentioned above. The UC-MSCs were loaded into the lower chambers, and when the confluence reached 80%, the BMSCs/HUVECs (1 × 10^4^ cells per well) were seeded in the upper chambers. After 24 hours, nonmigrating cells on the upper side of the membrane were wiped off, and cells that had migrated to the lower layer were fixed with methanol for 20 min and then stained with 0.1% crystal violet (Solarbio, Beijing, China) for 15 min. The stained cells were then observed under a light microscope (Nikon Ni-U, Japan) at 100x magnification. The number of migrated cells was counted within five randomly selected fields for each well.

*(3) Apoptosis Assay*. The one-step terminal transferase-mediated deoxyuridine triphosphate (dUTP) nick-end labeling (TUNEL) Kit (Beyotime, Shanghai, China) was used for detecting cell apoptosis. Briefly, the BMSCs/HUVECs were seeded into the lower chambers at a density of 6 × 10^4^ cells per well in a 12-well plate, whereas UC-MSC cells (3 × 10^4^) were placed in the inserts for both the UC-MSC and PRCR + UC-MSC groups and cultured in the serum-free medium for 96 hours in coculture systems as described above. Then, the cells were fixed with 4% paraformaldehyde for 20 min and incubated in 0.3% Triton X-100 solution for five min to permeabilize the cell membrane. After washing the cells with phosphate-buffered saline (PBS) three times, the cells were incubated with a reaction mixture at 37°C for 60 min. An antifade mounting medium with 4′, 6-diamidino-2-phenylindole (DAPI, Beyotime) was added to every well according to the manufacturer's protocol. Next, all nuclei were stained with DAPI while the TUNEL-positive nuclei were stained with green fluorescence. Fluorescent-stained cells were then observed and captured under a fluorescent microscope (Olympus IX 71, Japan) at 200x magnification. The number of apoptotic cells was counted within three randomly selected fields for each well.

*(4) Alkaline Phosphatase (ALP) and Alizarin Red (ALR) Staining Assay of BMSCs*. The BMSCs (4 × 10^4^ cells/well) were seeded in the lower chambers of a 24-well plate, whereas UC-MSC cells (2 × 10^4^ cells/well) were placed in the inserts for both the UC-MSC and PRCR + UC-MSC groups and cultured in the osteogenic differentiation medium (Cyagen, Guangzhou, China). The osteogenic differentiation induction medium was changed every 48 hours. After seven days of osteogenic induction, the BMSCs were fixed with 4% paraformaldehyde for 30 min and then stained with an ALP assay kit (Beyotime Biotechnology) for two hours. ALP activity was then measured with the BCIP/NBT Alkaline Phosphatase Color Development Kit (Beyotime Biotechnology). The ALP staining was subsequently observed and photographed under a light microscope (Nikon Ni-U, Japan) and evaluated using the ImageJ software (USA).

To evaluate the formation of the mineralized nodules, the BMSCs were stained with ALR solution (Cyagen) according to the manufacturer's protocol after 14 days of osteogenic induction. Each well was washed with PBS three times, and the staining results were observed under a light microscope (Nikon Ni-U, Japan). ImageJ was then used to quantify the percentage of the total area stained by ALR.

*(5) Tube Formation Assay of HUVECs*. HUVECs (6 × 10^4^ cells/well) were seeded onto 150 *μ*L growth factor reduced Matrigel (Corning, USA) coated 24-well plates, whereas the UC-MSC cells (3 × 10^4^ cells/well) were placed in the inserts for both the UC-MSC and PRCR + UC-MSC groups and cultured in the complete medium. After incubation for six hours, the capillary-like network was observed under a microscope (Olympus IX 71). The capacity of tube formation was quantified by calculating the number of tubes per field at 100x magnification. Three random fields were chosen per well for quantification using the ImageJ software.

*(6) Western Blotting Analysis*. Protein samples of BMSCs and HUVECs in different groups were extracted using the Minute Total Protein Extraction Kit (Invent Biotechnologies, USA) and diluted at a ratio of 1 : 4 with protein loading buffer (Epizyme Biotechnology, Shanghai, China). The content of each sample was determined by the bicinchoninic acid (BCA) protein assay kit (Thermo Scientific, USA). 30 *μ*g of protein was subjected to sodium dodecyl sulfate-polyacrylamide gel electrophoresis (SDS-PAGE) after denaturation at 95°C for ten min. After electrophoresis, the proteins were transferred to 0.22 *μ*m polyvinylidene difluoride membranes (Merck Millipore, Darmstadt, Hesse, Germany), blocked with protein-free rapid blocking buffer (EpiZyme) for 15 min, and subsequently reacted with the specific primary antibodies (1 : 1000) at 4°C overnight. After that, the membranes were washed three times in Tris Buffered saline Tween (TBST) and were incubated with horseradish peroxidase- (HRP-) conjugated secondary antibodies at 37°C for one hour. After the membranes were again washed with TBST three times, the bands were developed using Omni enhanced chemiluminescence (ECL) reagent (Epizyme) under e-Blot (Touch Imager, Shanghai, China). The gray intensity of the bands formed was quantified using the ImageJ software.

The primary antibodies used were anti-Runt-related transcription factor 2 (RUNX2, 1 : 1000, Affinity, AF5186), anti-VEGFA (1 : 1000, Abcam, ab46154), anti-B-cell lymphoma-2 (Bcl-2, 1 : 1000, Affinity, AF6139), anti-caspase-3 (Huabio, 1 : 1000, ET1608-64), and anti-GAPDH (1 : 5000, EpiZyme, LF205).

### 2.2. *In Vivo* Experiments

#### 2.2.1. Animal Model and Grouping

All animal experimental procedures were approved by the Sun Yat-sen University Animal Care and Use Committee (SYSU-IACUC-2020-000483) and performed following the animal care and ethical guidelines. A total of 40 specific pathogen-free (SPF) male Sprague-Dawley (SD) rats weighing 300 ± 20 g was purchased from Sun Yat-sen University. Rats were group-housed by three per cage in a temperature-controlled room at 25°C with 12 h light-dark cycles and access to food and water *ad libitum*. To establish the ONFH model, 2 intraperitoneal injections of lipopolysaccharide (LPS, 20 *μ*g/kg, Solarbio, Beijing) were administered at an interval of 24 hours, followed by three intramuscular injections of high-dose methylprednisolone (MPS, 40 mg/kg, Pfizer, Shanghai, China) for three consecutive days each week for three weeks. 40 rats were randomly assigned to five groups: (1) Control group (*n* = 8), normal rats that did not receive any intervention; (2) MPS group (*n* = 8), ONFH model rats with no treatment; (3) PRCR group (*n* = 8), ONFH model rats treated with 50 *μ*L PRCR without dilution via injection into each hip joint; (4) UC-MSC group (*n* = 8), ONFH model rats treated with an injection of UC-MSCs (5 × 10^5^ cells) resuspended in 50 *μ*L normal saline into each hip joint; (5) PRCR + UC-MSC group (*n* = 8), ONFH model rats treated with UC-MSCs (5 × 10^5^ cells) resuspended in 50 *μ*L PRCR without dilution via injection into each hip joint. For groups (3), (4), and (5), the PRCR or UC-MSCs were injected simultaneously into bilateral joints on the first day of the weekly MPS injection. The rats were anesthetized by intraperitoneal pentobarbitone sodium (2%, 2 mL/kg; Sigma-Aldrich) injections, and the intra-articular injections were guided by ultrasound (SONIMAGE HS1, Konica Minolta, Tokyo, Japan) according to the methods introduced in our previously published article [30]. The injections were performed using the in-plane technique with a 25G (Gastight 1810 Series, Hamilton Bonaduz AG, Bonaduz, Switzerland), and the real-time needle path was visualized. All the procedures were performed under aseptic conditions. Six weeks later, the rats were euthanized, and the femoral heads were examined by microcomputed tomography (micro-CT) and histomorphological analysis.

#### 2.2.2. Micro-CT Measurement

The femoral heads were harvested with excess soft tissues dissected, fixed in formalin, and analyzed by SkyScan1276 (Bruker MicroCT, Kontich, Belgium) to evaluate the trabecular bone morphology and structure of the femoral head. All the samples were scanned with the following parameters: source current, 200 *μ*A; source voltage, 85 kV; filter, AI 1.0 mm; and rotation step, 0.3 degrees. The scanning subchondral region of interest (ROI) within this volume was manually defined, and trabecular bone parameters including trabecular thickness (Tb.Th), trabecular separation (Tb.Sp), bone volume per tissue volume (BV/TV), and trabecular number (Tb.N) were quantified. The coronal, sagittal, and transverse sections of the samples from each group were generated using the DataViewer software (Bruker Micro-CT). In addition, the samples were decalcified for follow-up experiments.

#### 2.2.3. Hematoxylin and Eosin (H&E) Staining and Tartrate-Resistant Acid Phosphatase (TRAP) Staining

The femoral head samples were fixed using 4% paraformaldehyde, decalcified with 10% EDTA solution, embedded in paraffin, and then cut into 5 *μ*m sections with a microtome. The sections were deparaffinized with xylene and rehydrated in a graded series of ethanol solutions, before being stained with hematoxylin and eosin (H&E). H&E staining was performed to detect pathological characteristics and changes in the femoral heads from different groups, and the slices were observed under a light microscope (Nikon Ni-U, Japan).

TRAP staining was used to identify osteoclast cells of each group. The slides were incubated with TRAP staining solution (Servicebio, Wuhan, China) for one hour in the dark. The osteoclasts were defined as cells that are TRAP-positive with more than three nuclei. The apoptotic cells were detected with a TUNEL staining kit (Servicebio, Wuhan, China). Apoptotic cells appeared brown.

#### 2.2.4. Immunohistochemistry (IHC)

VEGFA and osteogenesis-related molecule expression were assessed by immunohistochemistry (IHC) using antibodies against VEGFA, RUNX2, and ALP on formalin-fixed paraffin-embedded (FFPE) tissue sections. The embedded samples were cut into 5 *μ*m sections, deparaffinized with xylene, rehydrated using graded ethanol, antigen-retrieved, and then incubated with specific primary antibodies at 4°C overnight, and biotinylated secondary antibodies were incubated at room temperature for one hour. Then, sections were visualized with DAB precipitate and counterstained with hematoxylin. Images were captured under a light microscope (Nikon Ni-U, Japan). The mean density of protein expression was evaluated using the ImageJ software (USA).

The primary antibodies were anti-VEGFA (1 : 100, Abclonal, A12303), anti-RUNX2 (1 : 100, Affinity, AF5186), and anti-ALP (1 : 100, ImmunoWay, YT5563). The secondary antibodies were Goat Anti-Rabbit IgG (H + L) HRP (1 : 200, Affinity, S0001).

### 2.3. Statistical Analysis

All *in vitro* experiments were repeated three times. Statistical analyses were performed using GraphPad Prism 8.0 (San Diego, CA, USA). Data were shown as mean ± standard deviation (SD). Differences among groups were assessed by one-way analysis of variance (ANOVA) with Bonferroni post hoc test for multiple-group comparisons. *P* values < 0.05 were considered statistically significant.

## 3. Results

### 3.1. Platelet and Growth Factor Concentration

Since apheresis platelets were adopted in this study; red blood cells (RBC) and leukocytes were almost depleted in the PRP mixture. Before activation, the mean concentration of platelets in the PRP was 1013.83 ± 93.14 × 10^9^/L. The results of ELISA showed that the average concentrations of TGF-*β*1 and VEGF-A in PRCR after the single freeze-thaw were 155.34 ± 42.06 ng/mL and 339.04 ± 66.75 pg/mL, respectively. The diluted PRCR was used for *in vitro* experiments and the undiluted PRCR for *in vivo* experiments.

### 3.2. Effects of Different Concentrations of PRCR on UC-MSC Proliferation

**Figure 1:**
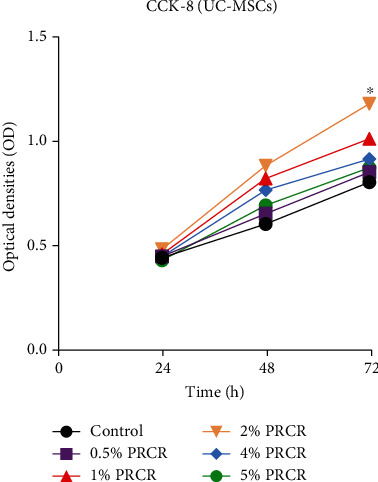
Effect of different concentrations of PRCR on UC-MSC proliferation. CCK-8 assay indicated that the UC-MSCs treated with 2% PRCR achieved maximum proliferation. *n* = 3; ∗*P* < 0.05, compared to the other group. PRCR: platelet-rich plasma clot releasate; UC-MSCs: umbilical cord mesenchymal stem cells; CCK-8: cell counting kit-8 assay.

To detect the optimum PRCR concentration for UC-MSC culture, different concentrations of PRCR were added into the culture medium. When cultured with a normal culture medium and different concentrations of PRCR, the UC-MSCs adherent to the culture dish showed a fibroblast-like, spindle-shaped morphology. Results from this study revealed that PRCR improved the proliferation of UC-MSCs in a time-dependent manner. Meanwhile, UC-MSCs treated with 2% PRCR exhibited significantly increased cell proliferation compared with the other group on day 3 (*P* < 0.05). No significant differences were detected in other concentration groups on day 1 or day 2 (Figure 1).

### 3.3. Effects of PRCR and UC-MSCs on DEX-Treated BMSC Proliferation, Migration, and Osteogenesis Differentiation *In Vitro*

**Figure 2:**
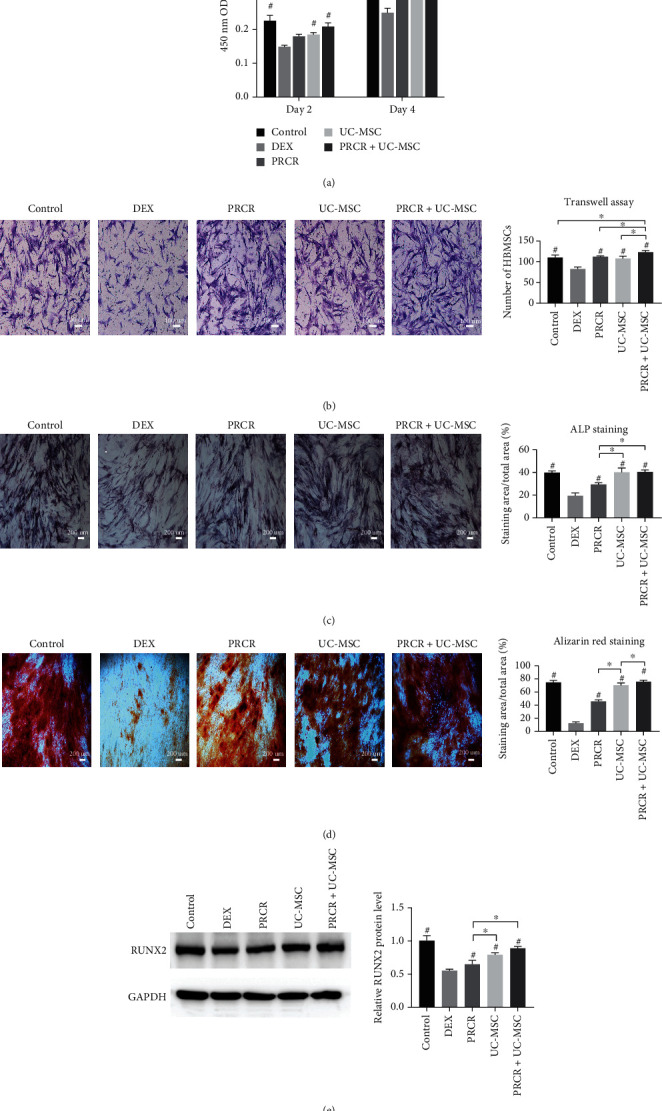
*In vitro* effects of PRCR, UC-MSCs, and PRCR + UC-MSCs on the proliferation, migration, and osteogenesis differentiation of DEX-treated BMSCs in the coculture systems. (a) Cocultured with PRCR, UC-MSCs, and PRCR + UC-MSCs, the cell viability of DEX-treated BMSCs was examined by CCK-8 assay. The treatment groups, including the PRCR, UC-MSC, and PRCR + UC-MSC groups, significantly promoted the BMSC proliferation inhibited by DEX (*P* < 0.05). (b) Migration activities of DEX-treated BMSCs were examined by Transwell assay in different coculture conditions, followed by quantitative analysis. The treatment groups significantly promoted the BMSC migration inhibited by DEX (*P* < 0.05). (c, d) Representative images of ALP staining (day 7) and ALR staining (day 14) under osteogenic medium in different coculture conditions, followed by quantitative analysis. The treatment groups significantly promoted the BMSC osteogenic differentiation inhibited by DEX (*P* < 0.05). (e) Western blotting was conducted to evaluate the RUNX2 expression. Higher levels of RUNX2 protein were detected in all the treatment groups compared to the DEX group (*P* < 0.05) (*n* = 5, ^#^Comparisons between the DEX group and other groups, *P* < 0.05; ^∗^Comparisons between the two groups, *P* < 0.05). PRCR: platelet-rich plasma clot releasate; UC-MSCs: umbilical cord mesenchymal stem cells; BMSCs: bone marrow mesenchymal stem cells; DEX: dexamethasone; CCK-8: cell counting kit-8 assay; ALP: alkaline phosphatase; ALR: alizarin red.

Previous studies have shown that as little as 10^−6^ M DEX inhibits BMSC proliferation and induces apoptosis for *in vitro* assays [31]. The DEX concentration adopted in this study was described in a previous study [26]. To evaluate the effects of PRCR and UC-MSCs on the viability and proliferation of DEX-treated BMSCs, a CCK-8 assay was performed. We found that 10 *μ*M DEX significantly inhibited the proliferation of BMSCs (*P* < 0.05), while the inhibitory effect was reversed by PRCR, UC-MSC, and PRCR + UC-MSC treatments (*P* < 0.05). On day 4, the PRCR + UC-MSC group demonstrated significantly promoted proliferation compared with the PRCR group and the UC-MSC group (*P* < 0.05) (Figure 2(a)). As for migration capacity, PRCR, UC-MSC, and PRCR + UC-MSC treatments significantly restored the impaired migration ability of DEX-treated BMSCs (*P* < 0.05). It was also revealed that the PRCR + UC-MSC group held greater cell migration potential than the PRCR group, the UC-MSC group, and the control group (*P* < 0.05) (Figure 2(b)). In the osteogenic induction assays, our results showed decreased ALP activity, and fewer calcium nodules formed in the DEX group, so it can be deduced that DEX remarkably inhibited the osteogenic differentiation of BMSCs. Upon further quantitative analysis, the combination of PRCR and UC-MSCs exhibited better osteogenic induction effects than the PRCR alone (*P* < 0.05) (Figures 2(c) and 2(d)). Higher levels of RUNX2 protein were detected in all the treatment groups compared to the DEX group (Figure 2(e)).

### 3.4. Effects of PRCR and UC-MSCs on DEX-Treated HUVEC Proliferation, Migration, and Angiogenesis *In Vitro*

**Figure 3:**
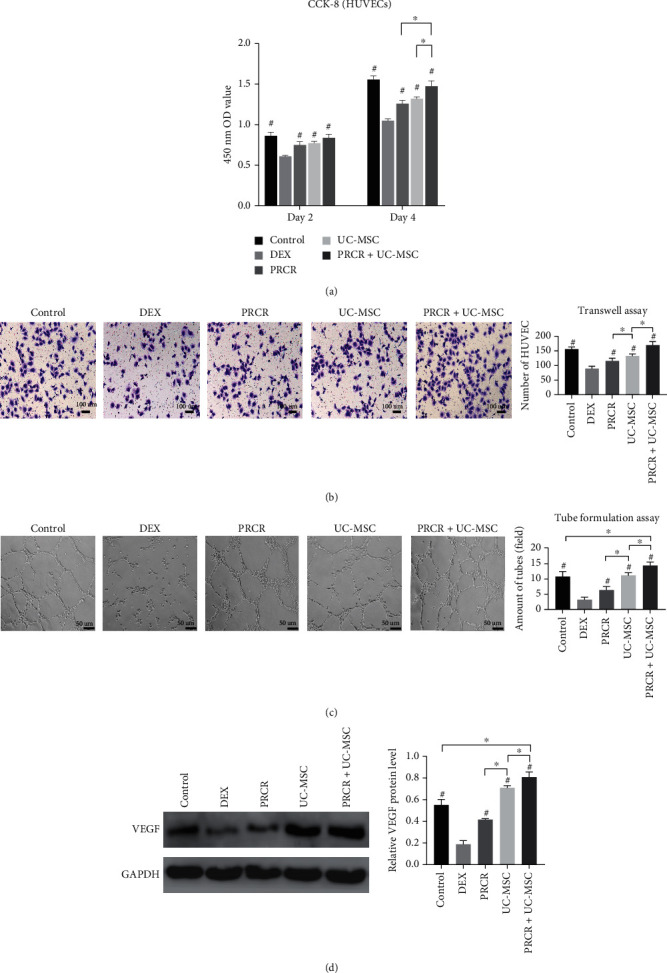
*In vitro* effects of PRCR, UC-MSCs, and PRCR + UC-MSCs on the proliferation, migration, and capillary-like tube formation of DEX-treated HUVECs in the coculture systems (a) Cocultured with PRCR, UC-MSCs, and PRCR + UC-MSCs, the cell viability of DEX-treated HUVECs was examined by CCK-8 assay. These three treatment groups showed significant promotion of the HUVEC proliferation inhibited by DEX (*P* < 0.05). (b) Migration activities of DEX-treated HUVECs were examined by Transwell assay in different coculture conditions, followed by quantitative analysis. The treatment groups showed significant promotion of the HUVEC migration inhibited by DEX (*P* < 0.05). (c) Representative images of capillary-like tube formation in different coculture conditions, followed by quantitative analysis. The treatment groups showed significant promotion of the HUVEC angiogenesis differentiation inhibited by DEX (*P* < 0.05). (d) Western blotting was conducted to evaluate the VEGFA expression. Higher levels of VEGFA protein were detected in all treatment groups compared to the DEX group (*P* < 0.05) (*n* = 5, ^#^Comparisons between the DEX group and other groups, *P* < 0.05; ^∗^Comparisons between the two groups, *P* < 0.05). CCK-8: cell counting kit-8 assay; VEGF: vascular endothelial growth factor; GADPH: glyceraldehyde-3-phosphate dehydrogenase; PRCR: platelet-rich plasma clot releasate; UC-MSCs: umbilical cord mesenchymal stem cells; DEX: dexamethasone; GADPH: glyceraldehyde-3-phosphate dehydrogenase.

Compared to the control group, the viability and proliferation capacities of HUVECs were attenuated when treated with DEX (*P* < 0.05). As shown by the results of the CCK-8 assay, the PRCR, UC-MSC, and PRCR + UC-MSC treatments restored the HUVEC viability impaired by DEX on days 2 and 4 (*P* < 0.05) (Figure 3(a)). Similar to the cell viability results, the inhibitory effects of DEX on migration and tube formation capacities were reversed by PRCR, UC-MSCs, and PRCR + UC-MSC treatments (*P* < 0.05), and quantification analysis demonstrated that more migrated HUVECs, and tube-shaped cells were observed in the PRCR + UC-MSC group compared to the application of PRCR or UC-MSCs alone (*P* < 0.05) (Figures 3(b) and 3(c)). Western blotting revealed downregulation of VEGFA expression in the DEX group, while higher levels of VEGFA were detected in the UC-MSC and PRCR + UC-MSC groups (Figure 3(d)).

### 3.5. Effects of PRCR and UC-MSCs on DEX-Treated BMSC and HUVEC Apoptosis *In Vitro*

**Figure 4:**
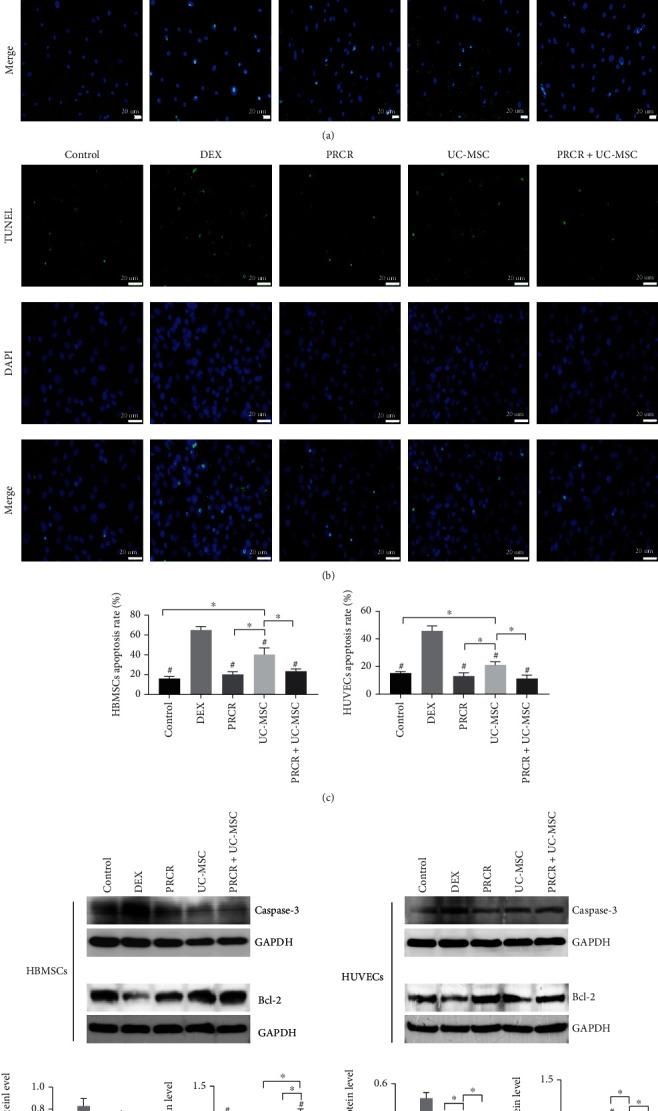
Effects of PRCR, UC-MSCs, and PRCR + UC-MSCs on DEX-treated BMSCs and HUVEC apoptosis. (a, b) Representative images of TUNEL-positive cells (apoptotic cells) of DEX-treated BMSCs and HUVEC apoptosis in different coculture conditions. (c) Quantitative analysis of apoptosis rates of BMSCs and HUVECs, respectively. The PRCR and PRCR+UC-MSCs rescued the DEX-induced apoptotic effects (*P* < 0.05). (d) Western blotting was conducted to evaluate the expression of the apoptosis-related proteins. The PRCR, UC-MSC, and PRCR + UC-MSC groups significantly blocked caspase-3 activation and increased Bcl-2 expression compared to the DEX group (*n* = 5, ^#^Comparisons between the DEX group and other groups, *P* < 0.05; ^∗^Comparisons between the two groups, *P* < 0.05). PRCR: platelet-rich plasma clot releasate; UC-MSCs: umbilical cord mesenchymal stem cells; DEX: dexamethasone; HBMSCs: human bone marrow mesenchymal stem cells; HUVECs: human umbilical vein endothelial cells; TUNEL: terminal transferase-mediated deoxyuridine triphosphate (dUTP) nick-end labeling; DAPI: 4′, 6-diamidino-2-phenylindole; GADPH: glyceraldehyde-3-phosphate dehydrogenase.

The antiapoptosis effects of PRCR, UC-MSC, and PRCR + UC-MSC treatments on DEX-treated BMSCs and HUVECs were evaluated by TUNEL assay. For both the BMSCs and HUVECs, the number of apoptotic cells in the DEX-treated groups was remarkably higher than the control group's (*P* < 0.05). Nevertheless, the applications of PRCR and PRCR + UC-MSCs reversed the DEX-induced apoptotic effects, respectively (*P* < 0.05). Moreover, the number of apoptotic cells was observed to significantly decrease in the PRCR group and PRCR + UC-MSC group compared to the UC-MSC group (*P* < 0.05) for both the BMSCs and HUVECs. Specifically, a higher apoptotic rate of BMSCs was observed in the UC-MSC group compared to the control group (*P* < 0.05) (Figures 4(a)–4(c)). In the DEX-treated BMSCs and HUVECs, the remarkable upregulation of caspase-3 and downregulation of Bcl-2 were confirmed. Different treatments applied to the coculture systems significantly blocked caspase-3 activation and increased Bcl-2 expression compared to the DEX groups (*P* < 0.05) (Figure 4(d)).

### 3.6. PRCR and UC-MSCs Rescued Subchondral Necrosis Changes and Bone Loss in ONFH Rat Model

**Figure 5:**
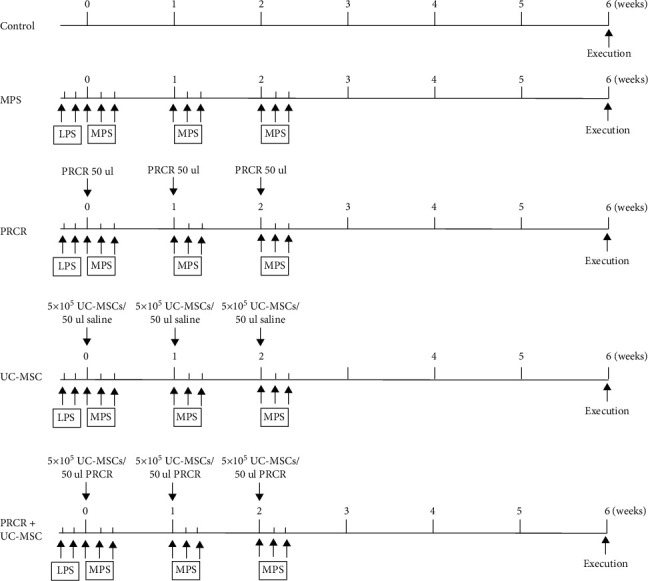
The time-course of animal experiments (ONFH model establishment and intra-articular injection schedule). ^∗^50 *μ*L injected for each hip joint. PRCR: platelet-rich plasma clot releasate; UC-MSCs: umbilical cord mesenchymal stem cells; LPS: lipopolysaccharide (LPS); MPS: methylprednisolone.

**Figure 6:**
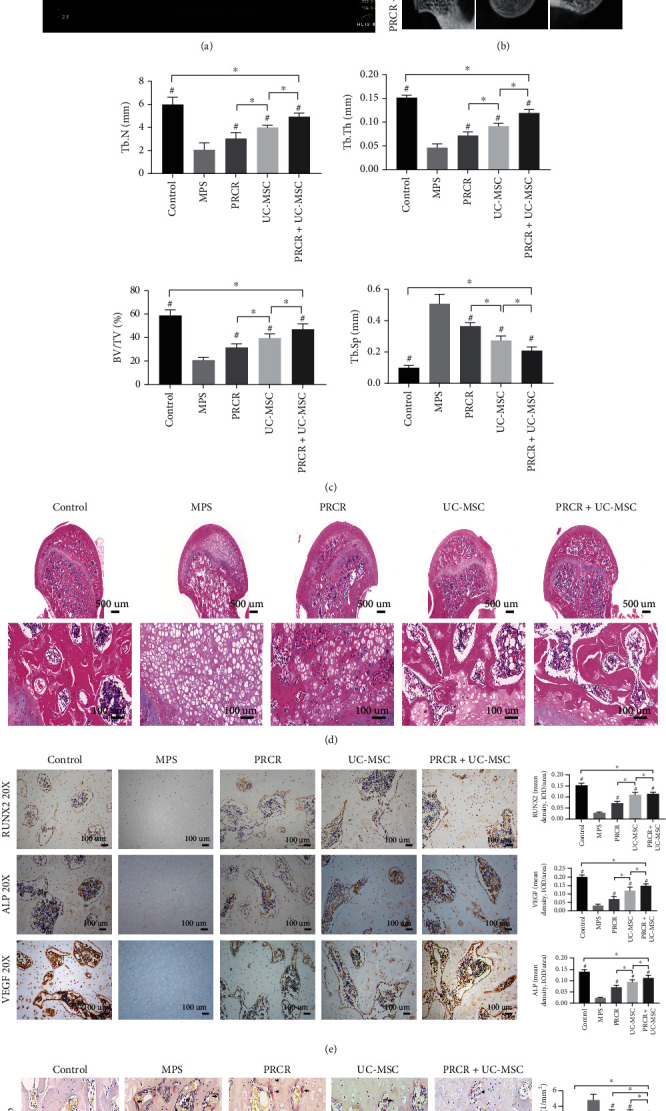
Effects of PRCR, UC-MSCs, and PRCR + UC-MSCs on bone tissue protection and necrosis restoration in the ONFH rat model. (a) Visualized intra-articular injection using ultrasonic guidance, the white arrows indicated the needle pathway. (b, c) Representative micro-CT images of femoral heads (coronal, transverse, and sagittal images) from different treatment groups followed by quantitative analysis. The PRCR, UC-MSC, and PRCR + UC-MSC treatments improved the microstructural parameters changes and bone loss compared to the MPS group (*P* < 0.05). (d) Hematoxylin and Eosin (HE) staining of femoral heads from different groups. (e) Immunohistochemical staining of RUNX2, ALP, and VEGF of different groups followed by quantitative analysis. The treatment groups significantly promoted the expression of RUNX2, ALP, and VEGF compared to the MPS group (*P* < 0.05). (f) Representative tartrate-resistant acid phosphatase (TRAP) staining images showing the distributions of osteoclasts in the femoral heads from different groups, followed by quantitative analysis. The black arrows indicate the osteoclasts. The number of TRAP-positive cells in the treatment groups was significantly lower compared to the MPS group (*P* < 0.05). (g) Representative TUNEL staining images of femoral heads from different groups, followed by quantitative analysis. The apoptotic cell numbers in the treatment groups were significantly decreased compared to the MPS group (*P* < 0.05) (*n* = 5 for each group. ^#^Comparisons between the DEX group and other groups, *P* < 0.05; ^∗^Comparisons between the two groups, *P* < 0.05). Tb.Th: trabecular thickness; Tb.Sp: trabecular separation; BV/TV: bone volume per tissue volume; Tb.N: trabecular number; PRCR: platelet-rich plasma clot releasate; UC-MSCs: umbilical cord mesenchymal stem cells; DEX: dexamethasone; TUNEL: terminal deoxynucleotidyl transferase-mediated deoxyuridine triphosphate (dUTP) nick-end labeling; VEGF: vascular endothelial growth factor; ALP: alkaline phosphatase; MPS: methylprednisolone.

To evaluate the effects of PRCR and UC-MSCs on MPS-induced ONFH, the injection treatments were performed according to the protocol mentioned above (Figure 5). All the intra-articular injections were performed under ultrasonic guidance (Figure 6(a)), and the protective effects of the treatments were confirmed by micro-CT analysis and HE staining. In the MPS group, trabecular bone loss was observed in subchondral areas. In contrast, the subchondral areas of the femoral heads appeared relatively intact upon treatments with PRCR, UC-MSCs, and PRCR + UC-MSCs. Further quantitative analysis indicated that the parameters of micro-CT including Tb.Th, BV/TV, and Tb.N in the MPS group were significantly reduced in the MPS group compared to the control group (*P* < 0.05), whereas the low bone mass induced by the high dose of MPS was improved by the intra-articular injections of PRCR, UC-MSCs, and PRCR + UC-MSCs (*P* < 0.05) (Figure 6(b) and 6(c)). As for histological changes, HE staining showed empty lacunae, sparse trabecular bone, and even subchondral microfracture in the MPS group, while the slices from the PRCR, UC-MSC, and PRCR + UC-MSC-treated groups showed fewer empty lacunae and less necrotic tissues (Figure 6(d)). IHC staining results revealed that the applications of PRCR, UC-MSC, and PRCR + UC-MSC treatments significantly increased the expression of RUNX2, ALP, and VEGF compared to the MPS group, even though these treatments failed to reverse the downregulation of osteogenesis and angiogenesis proteins compared to the control group (*P* < 0.05) (Figure 6(e)).

TRAP staining was performed to investigate whether intra-articular treatments influenced the osteoclast activities in the affected femoral heads. The number of TRAP-positive cells was higher in the MPS group compared to the control group, and the number of TRAP-positive cells for the intra-articular treatment groups was significantly lower than the MPS group (*P* < 0.05) (Figure 6(f)). TUNEL assay revealed that the number of apoptotic cells in the MPS group was remarkably increased whereas the MPS-induced apoptotic effect was attenuated by the intra-articular injections of PRCR, UC-MSCs, and PRCR + UC-MSCs (Figure 6(g)).

## 4. Discussion

High-dose glucocorticoids, such as methylprednisolone and dexamethasone, usually impair the natural healing potential of autologous bone-forming stem cells, which further inhibit bone reconstruction and new blood vessel formation. Therefore, it is well-accepted that glucocorticoid-induced ONFH is a disorder involving the abnormal processes of osteogenesis and angiogenesis [3, 6]. Currently, no single intervention can reverse the changes of necrosis, because none of them can target all the mechanical and biological types of damage involved in ONFH. Hence, effective treatment options that are minimally invasive and safe are still urgently needed for ONFH. Despite some favorable outcomes of pilot studies involving biological therapies, evidence supporting the combined use of PRP and MSCs for treating ONFH is still limited [19, 21]. This study suggests for the first time via its *in vitro* and *in vivo* results that the combination of PRCR and UC-MSCs reversed a series of glucocorticoid-induced responses, including decreased cell viability, inhibited migration, reduced differentiation capacities, and necrotic changes with bone mineral loss. In particular, the BMSCs and HUVECs induced with DEX in the *in vitro* studies were used to mimic the impaired microenvironment of the necrotic femoral head cavity that later interact with the PRCR and UC-MSCs *in vivo*. The current research is aimed at clarifying the cellular and molecular mechanisms involved in the therapeutic effects of PRCR and UC-MSCs on ONFH. The UC-MSCs have been evaluated to be viable for allogeneic clinical applications due to low immunogenicity [32], and the patient's autologous PRP (plasma component) will likely not reject its autogenous tissue microenvironment. According to our current PRCR preparation protocol, the erythrocyte and leukocyte concentrations in apheresis platelets were relatively low, which caused little influence on the immune response. Moreover, the autologous PRP is commonly applied in clinical practice, which is generally safe and effective for musculoskeletal disease management. The current results have led to speculation that combined biological therapies might be helpful for ONFH treatment, implying the possibility of clinical translation. Whether this hypothesis is correct is open to debate, but the findings from this study may shed light on future intra-articular therapies for ONFH. Notably, despite the promising results of *in vitro* and *in vivo* studies, the biological sustainability of UC-MSCs and PRP combined therapy was not directly verified in the current study. Thus, it is not appropriate to speculate on the certainty of the combination of PRP releasate and UC-MSCs for clinical practice, and results should be interpreted conservatively.

Both PRP and MSCs have shown considerable potential in regenerative medicine, and they share many common mechanisms in promoting bone reconstruction and angiogenesis [33]. Growth factors in PRP orchestrate MSC activities, such as proliferation, migration, and differentiation, which indicated that the benefits of transplanted MSCs are partially due to the paracrine effects stimulated by PRP [34]. Results from this study confirmed that the combined application of PRCR and UC-MSCs seemed to achieve synergistic effects since the PRCR + UC-MSC treatment was more effective than the ones with PRCR or UC-MSCs alone, both *in vitro* and *in vivo*. Generally, supplementary UC-MSCs in healthy states are prerequisites for cell-based biological therapies. PRCR assisted to stimulate the beneficial properties of UC-MSCs, which aids to explain why the combined application of PRCR and UC-MSCs would yield synergistic effects as demonstrated in our study. Although previous studies have demonstrated the promising prospects of MSC treatment for musculoskeletal disease, disadvantages including animal serum contamination, rapidly decreasing numbers, and the low survival rate of cells following a single injection hampered further clinical application [9, 35]. In contrast, both the PRP and PRP formulation possess the following advantages as potential candidates in bioactive therapy. Firstly, PRP can be used as an alternative safe serum source for the cultivation of UC-MSCs. Secondly, PRP contains various growth factors, cytokines, chemokines, and enzymes, which help to optimize the microenvironment needed for tissue healing [36]. Previous studies have proven that PRP promotes the proliferation of MSCs and does not interfere with lineage differentiation potential [36]. Lastly, data from this study also support the positive roles of PRCR on ONFH recovery, even when applied alone. Based on experimental feasibility, PRCR was chosen for this study, because the *in vitro* and *in vivo* studies could not be completed at the same time, and the biological activities of PRP after the freezing and thawing processes might have been compromised due to the degranulation process. Thus the PRP releasate was chosen for this study for homogenization. More importantly, the growth factors fully released in the PRCR needed to be quantified and used for concentration adjustment. We hope that the PRCR and growth factor concentration could provide an important reference for future studies. We chose a rat model for the preclinical *in vivo* studies. The circulating blood volume in rodents is approximately 55 to 70 mL/kg, and peripheral venous blood collection does not allow sufficient blood samples for the rat PRP autologous preparation. Thus, autologous transplantation was not feasible under the current study design. It should be acknowledged that autotransplantation could be a more safe and more immune-privileged method in future clinical practice.

Consistent with previous research, it was also demonstrated in this study that the growth factors in activated PRP could enhance the proliferation of UC-MSCs, and PRCR qualifies as a promising vehicle for MSCs in biological therapy [25]. TGF-*β* plays a significant role in promoting osteogenesis, angiogenesis, and MSC chemotaxis, and osteoblasts themselves are enriched with TGF-*β* receptors [37]. VEGF has been shown to be a key inducer of angiogenesis by regulating endothelial cell proliferation, migration, and vascularization. The CCK-8 assay indicated that the UC-MSCs treated with 2% PRCR achieved maximum proliferation. The current study revealed that the concentration of PRCR was related to the survival and proliferation of UC-MSCs, which was not totally concentration-dependent. Similar results were reported by a previously published study, and the 100% PRP *in vitro* may even cause marked toxicity to the cells [38]. Next, activated PRP was investigated on its ability to enhance the efficacy of UC-MSCs in treating DEX-induced BMSCs. Besides cell viability and apoptosis, the influence of DEX on migration capacity, which has rarely been discussed, was also explored. It is well-accepted that the migration ability of BMSCs is important for the maintenance of bone homeostasis since BMSCs can migrate to the necrotic lesions and differentiate into bone substances, especially under high-dose glucocorticoid conditions [39]. In this study, both PRCR and UC-MSCs were noticed to induce a significantly higher number of DEX-treated BMSCs to migrate across the Transwell membrane. Interestingly, a remarkably higher number of migrated BMSCs was observed in the PRCR + UC-MSC group than in the control group. One reasonable explanation would be the synergy between PRCR and UC-MSCs which exerted strong chemotactic effects on BMSCs.

Angiogenesis and osteogenesis are generally tightly coupled. Thus, angiogenesis is also pivotal throughout the entire process of bone reconstruction. Impaired migration and tube formation capacities are evidence of abnormal angiogenesis, and the harmful effects of DEX on endothelial cells have been discussed in previous studies [40, 41]. In this study, PRCR and UC-MSCs were proven to reverse the decreased viability and migration activity of HUVECs induced by DEX. Moreover, all the treatments induced an obvious positive effect on capillary-like tube formation, indicating enhanced angiogenesis. Previous studies have proven that the endothelial damage induced by DEX usually leads to reduced expression of VEGF, which directly affects angiogenesis and endothelial permeability. VEGF acts as the endothelial cell mitogen and an important angiogenic factor, while vascular endothelial growth factor A (VEGFA) is a specific key regulator of physiological angiogenesis. Data from this study showed that the PRCR, UC-MSC, and PRCR + UC-MSC treatments successfully rescued the inhibited VEGFA expression induced by DEX. In another disease model, Myung et al. demonstrated that PRP improved the therapeutic efficacy of UC-MSCs by enhancing their secretion of angiogenic factors, and increased expression of VEGF was also observed in the cotreated mice [42]. However, the detailed mechanism of PRP formulation and UC-MSCs combined therapy in promoting ONFH osteogenesis and angiogenesis needs to be further elucidated.

The therapeutic effects of PRCR + UC-MSC in promoting bone reconstruction in the ONFH rat model are of particular interest. The typical histological and radiological abnormalities and downregulation of ALP, RUNX2, and VEGF expression induced by high-dose MPS were observed in the model group. Additionally, TRAP and TUNEL staining showed that higher numbers of osteoclasts and apoptotic cells were localized in the necrotic area. These results indicate that decreased osteogenic and angiogenic-related protein expressions combined with an increase in osteoclast and apoptotic activities coincide with the rapid progression of ONFH. PRCR + UC-MSC treatment inhibited the osteoclasts and apoptotic activities in addition to upregulating the expressions of key proteins in ONFH restoration. Moreover, combined UC-MSC + PRCR treatment provided better outcomes than the treatment with PRCR or UC-MSCs alone upon a six-week follow-up period. PRP formulation has been proven to be effective in promoting bone regeneration and remodeling. For example, Tao et al. demonstrated that PRP-Exos play a positive role against endoplasmic reticulum stress-induced cell apoptosis and bone necrosis in ONFH [19, 26]. Preliminary studies have also been conducted to explore the use of UC-MSCs in treating ONFH. Chen et al. treated nine eligible patients at Association Research Circulation Osseous (ARCO) Stage II-IIIa with the intra-arterial infusion of UC-MSCs, and MRI evaluations showed that the UC-MSCs migrated into the necrotic tissue and further differentiated into osteoblasts to promote bone regeneration and reconstruction [19].

Results from our study are generally consistent with previous studies, since all the injection treatment groups showed significant improvements when compared with the MPS group, even though they were unable to completely reverse the pathological changes of ONFH. Based on the research results stated above, it is reasonable to suggest that the combined intra-articular application of PRCR and UC-MSCs might achieve improved clinical outcomes in patients with ONFH. Furthermore, special attention should be paid to the efficiency of MSCs and PRP formulation injection. It had been proved that the combinatorial therapy of MSC core decompression played a positive role in treating ONFH [43]. In this present study, PRCR and UC-MSCs were delivered to the hip joints via ultrasound-guided intra-articular injections, which possesses many advantages, such as real-time visualization, safety, and portability [30, 44]. Compared to intravascular infusion, *in situ* delivery of MSCs could overcome the obstacles of insufficient cells and difficulty in tracing the distribution of MSCs. It is obvious that the intra-articular PRP and UC-MSCs could not reach the subchondral tissue directly, and we speculate that the intra-articular treatments might influence the subchondral environment positively in ways that are not yet fully known. Several possible mechanisms have been considered. First, the MSCs and PRP might protect the necrotic femoral head by a paracrine mechanism, and the secreted growth factors and cytokines further promoted the subchondral osteogenesis and angiogenesis. Second, intra-articular therapy helps to dilate the local blood vessels and decrease blood viscosity, which further improves the blood circulation in the affected femoral heads and improved tissue hypoxia. We hypothesize that the positive effects might be due to the high local concentration of MSCs contacting the hip cavity directly. Up till now, the optimum number of intra-articular injected MSCs remained unclear, with cell numbers ranging from 10^6^ to 10^9^ for clinical ONFH patients, as reported in previous studies. A dose conversion was performed based on the volume of the joint cavity in rats, and 5 × 10^5^ was determined to be the final number of injected cells [45]. Despite all these findings, there is still an urgent need for well-designed clinical trials to comprehensively and systematically confirm the effectiveness and safety of this combinatorial biological therapy. It should be emphasized that regenerative medicine is still posing challenges, instead of being accepted as the usual treatment of ONFH. In particular, some promising subchondral and bone histological and molecular biologic changes were observed after treatment. Unfortunately, this present study and previous clinical studies all failed to provide direct evidence about the influence of intra-articular injections on the deep structures (for example, the bone marrow). We speculate that the therapeutic effects on the in situ microenvironment have profoundly influenced multiple signaling pathways [46], activating a series of downstream cascade reactions. In the future, more effort should be put into identifying the correlation between these factors, thus providing more direct evidence.

There are several limitations to this present study. First of all, the current results were inconclusive on whether the restorative osteocytes resulted from the differentiation of transplanted UC-MSCs or from their paracrine effects on BMSCs. Secondly, since there are a lack of robust *in vivo* cell tracing techniques, following the dynamic changes of UC-MSCs intra-articular distribution remains a challenging task that has yet to be accomplished. Changes within the microenvironment might be multifactorial, and the miroenvironment of the joint with ONFH is complex. To date, there is still no single perfect cell model which can mimic the clinical disease completely. Additionally, data from this current study are not sufficient to directly explain the reasons behind the improvement of subchondral structures upon intra-articular injections. Furthermore, the freeze/thaw and cryopreservation of PRCR were inevitable in this study, and the influence of these processes on the concentrations of the growth factors in PRCR is worth further studying. Lastly, early diagnosis and timely treatment of ONFH are critical for promising outcomes. However, the injection protocol in this study does not coincide with conventional treatment timing. Notably, few patients would choose to receive injection treatment before the onset of symptoms indicating ONFH; thus, these results in this study must be interpreted with caution in guiding clinical practice.

## 5. Conclusion

This is the first integrated study focusing on the combinatorial use of PRCR and UC-MSCs during the progression of ONFH. Our study suggests positive, synergistic effects of UC-MSCs and PRCR in treating ONFH. PRCR combined with UC-MSCs could effectively rescue DEX-induced apoptosis *in vitro* and promote the restoration and regeneration of necrotic bone *in vivo*. Furthermore, liquid injectable PRCR and UC-MSCs could be applied in a minimally invasive way, shedding light on future strategies for the clinical management of ONFH.

## Data Availability

The data used to support the findings of this study are available from the corresponding authors upon request.
